# Supplementary material for: All-magnonic repeater based on bistability
Source: Nat Commun. 2024 Aug 31;15:7577. doi: 10.1038/s41467-024-52084-0 (PMC11365973; doi:10.1038/s41467-024-52084-0)
Supplement: Supplementary file 1 — Supplementary Information [file 41467_2024_52084_MOESM1_ESM.pdf]

# Supplementary Materials
# All-magnonic repeater based on bistability

*Qi Wang*[1], *Roman Verba*[2], *Kristýna Davídková*[3], *Björn Heinz*[4], *Shixian Tian*[5], *Yiheng Rao*[5,6], *Mengying Guo*[1], *Xueyu Guo*[1], *Carsten Dubs*[7], *Philipp Pirro*[4], *Andrii V. Chumak*[3]

[1] *School of Physics, Huazhong University of Science and Technology, Wuhan, China*

[2] *Institute of Magnetism, Kyiv, Ukraine*

[3] *Faculty of Physics, University of Vienna, Vienna, Austria*

[4] *Fachbereich Physik and Landesforschungszentrum OPTIMAS, Rheinland-Pfälzische Technische Universität Kaiserlautern-Landau, Kaiserslautern, Germany*

[5] *School of Microelectronics, Hubei University, Wuhan, China*

[6] *Hubei Yangtze Memory Laboratories, Wuhan, China*

[7] *INNOVENT e.V., Technologieentwicklung, Jena, Germany*

## 1. Simulated Foldover and Bistability

In the main manuscript, there are several small peaks in the BLS spectra visible (see Fig. 2), which are attributed to the higher width modes and can be ignored in our studies. To verify this, we performed micromagnetic simulations similar to the experiments, where the excitation frequency was swept from 4.3 GHz to 8.0 GHz (or vice versa) with a step size of 50 MHz. To excite spin waves, we first calculate the Oersted field distribution of a 2 μm wide strip antenna with current of 20 mA in the magneto-static approximation and plug it into Mumax$^3$ with a varying microwave frequency $f$. The $M_y(x,y,t)$ of each cell was collected over a period of 100 ns and recorded in 50 ps intervals. The oscillations $m_y(x,y,t)$ were calculated for all cells via $m_y(x,y,t) = M_y(x,y,t) - M_y(x,y,0)$, where $M_y(x,y,0)$ corresponds to the ground state. The spin-wave intensity was extracted 5 μm far from the antenna. All the simulations were performed with a temperature of 300 K.

Figure S1(a) shows the simulated spin-wave spectra with a foldover effect and bistable window similar to the experimental results. Several small peaks are also observed in the simulations corresponding to the higher width modes with one or two orders of magnitudes smaller intensity. Interestingly, the observed higher-order modes are the 7$^{th}$, 9$^{th}$ and 11$^{th}$ modes, as confirmed by the comparison with a linear excitation spectrum at 10 times smaller driving field (see the red dashed line in Fig. S1(a)) and the simulated dispersion curve as show in Fig. S2. Simultaneously, in the nonlinear excitation regime we don't observe the excitation of 3$^{rd}$ and 5$^{th}$ modes (even modes have zero overlap with rf field and cannot be excited).

We verified the excitation of the fundamental mode in the nonlinear regime. Figure S1(b) show the simulated spin-wave amplitude distribution for a frequency of 6.9 GHz along the waveguide for the case of the frequency upsweep. It is clear that only the fundamental width mode is excited. A corresponding experiment is performed by sending a microwave current of frequency 6.9 GHz at power of 15 dBm to the pump antenna to directly excite propagation spin waves. The BLS is used to measure the spin-wave intensity across the width of the waveguide as shown in Fig. S1(c). A sinusoidal shape of the profile is obtained indicating the fundamental mode. The same, fundamental mode was the only observed at other points at the frequency upsweep path until 7.5 GHz, where the excitation drops.

Suppression of higher-order modes in the nonlinear regime is quite simple in nature. As explained in [S1], under a deeply nonlinear excitation, the nonlinear ferromagnetic resonance frequency under the antenna is shifted to match the excitation frequency. Similarly,

the frequencies of the higher-order width modes also shift up, and the whole higher-order branch lies above the excitation frequency. Thus, only the fundamental mode survives. This is another intriguing advantage of the deeply nonlinear excitation mechanism. In contrast, in the linear mode, multiple mode can be excited in a wide waveguide.

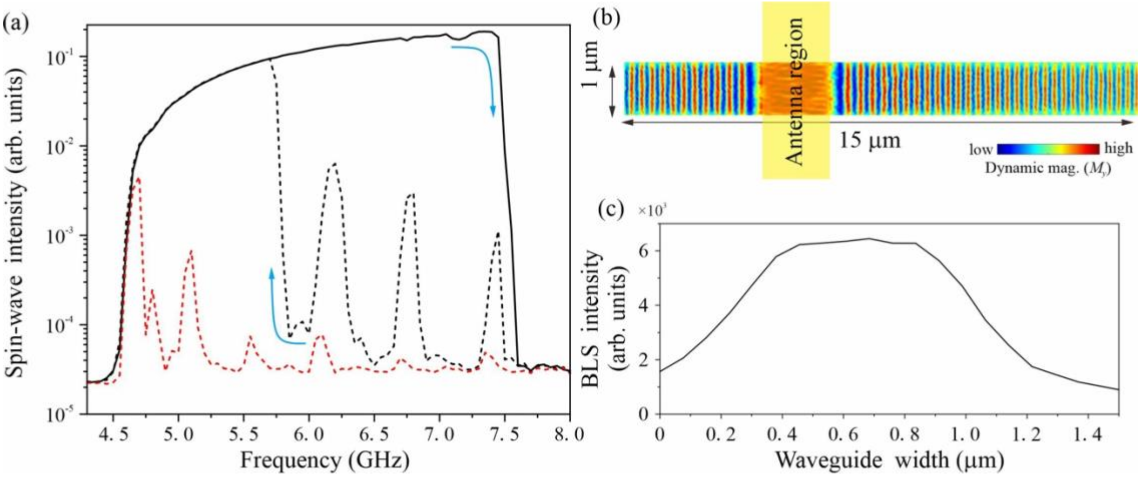


*Fig. S1* ***Simulated Foldover effect and Bistability****. (a) Simulated spectra show a foldover effect and large bistable window. Red dashed line shows the down-sweep curve with 10 times smaller driving field. (b) The simulated spin-wave amplitude distribution (frequency of 6.9 GHz) along the waveguide for the case of up frequency sweep. (c) The BLS intensity of frequency 6.9 GHz (Power P=15 dBm) across the width of 1 μm wide waveguide.*

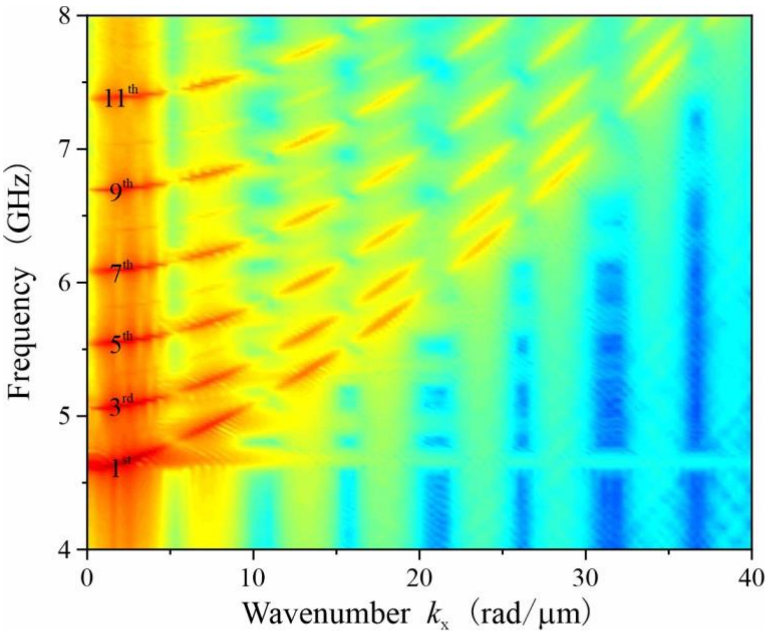


*Fig. S2 Simulated dispersion curve of 1 μm wide waveguide.*

## 2. Influence of the Source and Pump Power

Figure S3 shows the influence of source and pump power on the spin-wave intensity for the case of source + pump with the same period as shown in Fig. 3(c) in the main manuscript. Figure S3(a) and (b) show the experimental results of the integrated spin-wave intensity as function of time for different (a) source powers (pump power @10 dBm) and (b) pump powers (source power @6 dBm). It can be seen that the spin-wave intensity is almost constant once the source or pump power are above their respective power thresholds, indicating a stable and large operating window. Only the switching time changes as the source and pump powers are varied. The simulated results shown in Fig. S3(c) and (d) reproduce this phenomenon well.

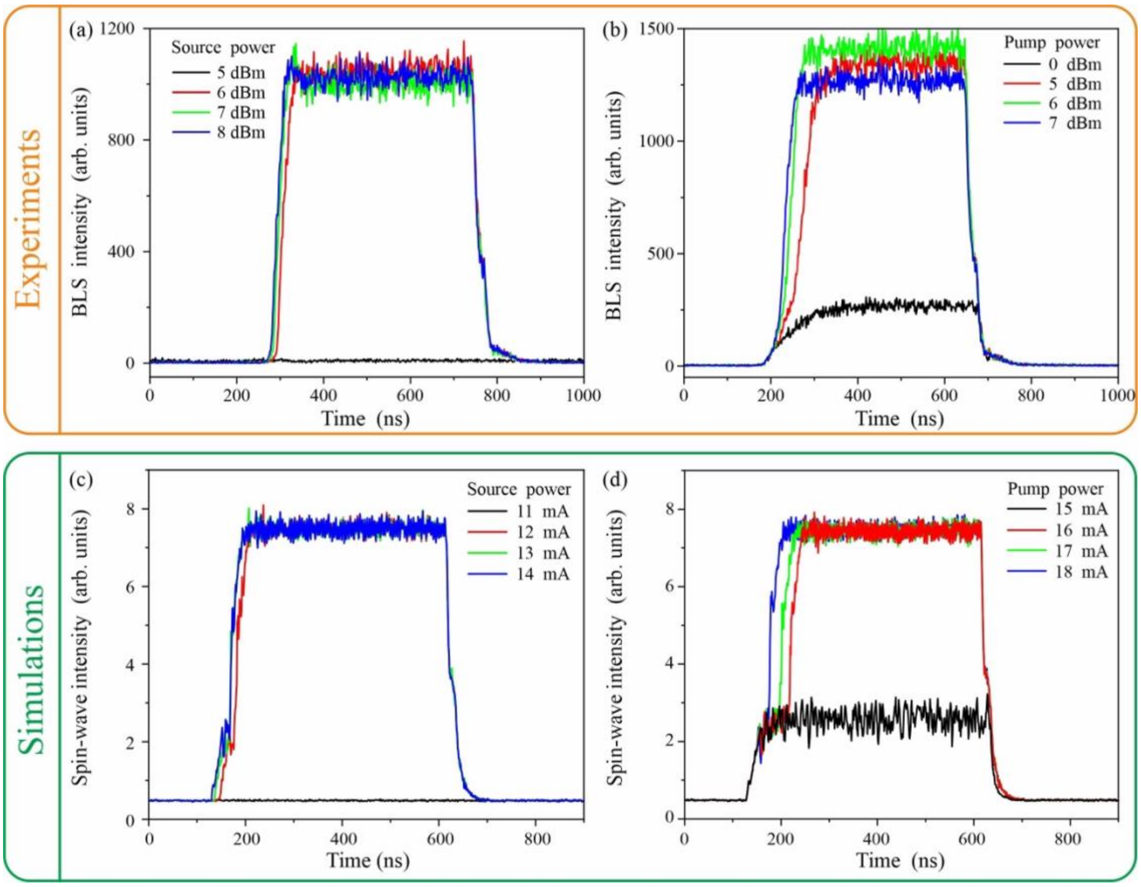


*Figure S3.* ***Influence of source and pump power.*** *The integrated spin-wave intensity as function of time for different (a) source powers (pump power @10 dBm) and (b) pump powers (source power @6 dBm). The simulated spin-wave intensity for different (c) source powers (pump power @20 mA) and (d) pump powers (source power @15 mA).*

## 3. Magnonic Repeater with Different Materials and Sizes

In order to prove its universality, additional micromagnetic simulations were performed with different materials and varying size. Figure S4 shows the simulated spin-wave intensity of three different cases: source only, pump only and source + pump for (a) 100 nm wide and 44 nm thick YIG waveguide with working frequency of 8.8 GHz, (b) 50 nm wide

and 5 nm thick CoFeB waveguide with working frequency of 61 GHz. The results are similar to Fig. 3 in the main text: the spin-wave intensity is amplified once the source and pump are simultaneously applied which indicates that the bistable switching using a propagation spin wave is a universal method in magnonics for different materials and varying sizes.

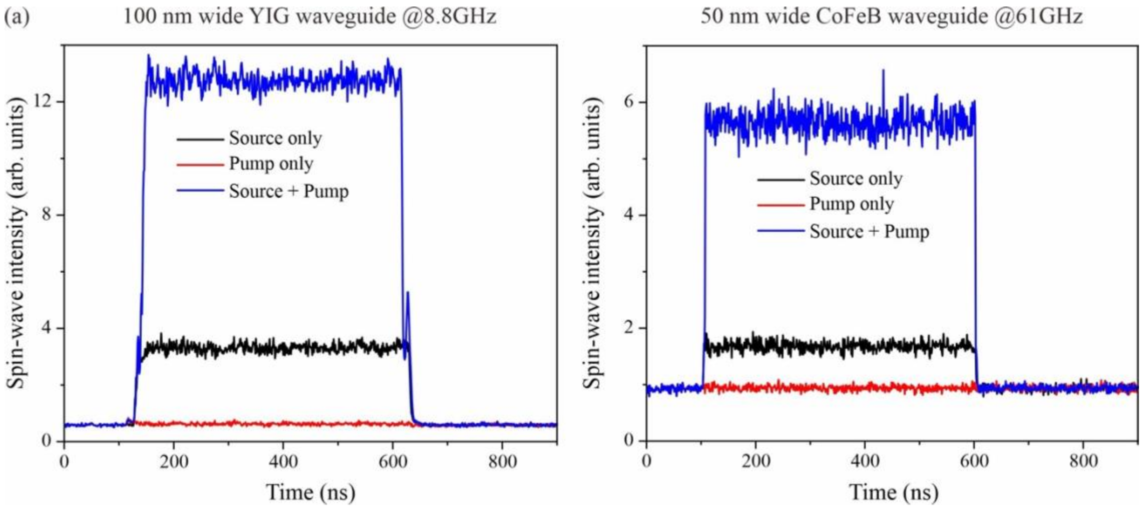


*Fig. S4* ***Influence of different materials and varying sizes.*** *The simulated spin-wave intensity of three different cases: source only, pump only and source + pump for (a) 100 nm wide and 44 nm thick YIG waveguide with excitation frequency of 8.8 GHz, (b) 50 nm wide and 5 nm thick CoFeB waveguide with excitation frequency of 61 GHz.*


[S1] Wang, Q.et al. Deeply nonlinear excitation of self-normalized short spin waves, Sci. Adv. 9, eadg4609 (2023)
